# Design and Performance Validation of a Low-Loss Optical System with Light Source Fluctuation Compensation for In Situ Deep-Sea Nitrate Sensing

**DOI:** 10.3390/s26196019

**Published:** 2026-09-23

**Authors:** Zhanfei Zhang, Jing Dong, Yahui Li, Yafeng Wu, Guojun Wu

**Affiliations:** 1 Xi’an Institute of Optics and Precision Mechanics, Chinese Academy of Sciences, Xi’an 710119, China; zhangzhanfei@opt.ac.cn (Z.Z.);; 2 Laoshan National Laboratory, Qingdao 266237, China

**Keywords:** in situ measurement, UV spectrophotometry, nitrate concentration, Argo float, low loss, dual beam

## Abstract

In situ measurement of deep-sea nitrate concentration is of significant importance for marine environmental monitoring and management. Nitrate sensors based on ultraviolet (UV) spectrophotometry, featuring simple, rapid, and reagent-free detection, are highly suitable for long-term autonomous monitoring and have attracted increasing research interest. However, existing systems still suffer from increased measurement errors and reduced detection ranges due to light source attenuation and optical transmission losses. To address these limitations, this study presents an optical system for a deep-sea nitrate sensor designed for deployment on mobile platforms. A co-aperture coupling design integrating the measurement and reference paths is proposed to achieve low optical loss while accurately compensating for the impact of light source attenuation on measurement accuracy. Furthermore, full-UV optical component optimization is performed to significantly improve optical energy utilization. Light-source calibration experiments demonstrate stable absorbance responses and effectively compensate for measurement deviations caused by light source attenuation. Performance evaluation of the system demonstrated a detection limit of 0.0056 mg/L-N(0.4 µmol/L, 10 mm optical path), with high measurement accuracy and precision across samples with varying nitrate concentrations. Sea trial results confirmed that the proposed system satisfies the requirements for stability and reliability in deep-sea in situ nitrate concentration monitoring, highlighting its potential for practical marine applications.

## 1. Introduction

Nitrate is one of the primary nutrients contributing to marine eutrophication [1]. Excessive nitrate concentrations can induce harmful algal blooms (HABs), including red tides and green tides, which severely disrupt marine ecosystems [2,3]. As an essential nutrient for the growth of aquatic plants, including phytoplankton [4,5], nitrate also affects the distribution and abundance of various algal species and other marine organisms [6], representing a crucial component of the marine nitrogen cycle [7]. Moreover, marine nitrate serves as an indicator of climate change [8,9], directly or indirectly influences the health and productivity of fishery resources [10,11], and provides important references for water quality assessment [12,13]. Therefore, monitoring seawater nitrate concentrations is of great significance for maintaining marine ecosystem health [14], protecting the marine environment, supporting sustainable fisheries development, addressing climate change, and ensuring drinking water safety.

Numerous studies have been conducted on nitrate detection, which can be generally categorized into three main approaches: wet chemical methods, electrochemical methods, and spectrophotometric methods. Wet chemical methods are simple in principle and cost-effective but suffer from limited sensitivity and low detection efficiency. Electrochemical methods provide rapid response and high sensitivity; however, they are susceptible to environmental interference and exhibit relatively poor stability. Spectrophotometric methods, featuring high sensitivity, good selectivity, and rapid detection capabilities, have become one of the most actively investigated approaches [15]. Among them, nitrate sensors based on ultraviolet (UV) spectrophotometry determine nitrate concentrations by analyzing the absorption spectra of water samples. These sensors require no chemical reagents, offer advantages of miniaturization and portability, and are highly suitable for in situ detection. The detection process is simple, rapid, and cost-effective, thereby attracting increasing research interest.

In 1999, Collos et al. proposed a reagent-free and rapid optical method for determining micromolar nitrate concentrations in seawater algal culture media based on UV absorption spectroscopy [16]. The method determined nitrate concentrations by measuring sample absorbance at a single wavelength of 220 nm, achieving a detection limit of 1 μmol/L and a precision of 1.5%. In 2002, Johnson and Coletti developed the In-Situ Ultraviolet Spectrophotometer (ISUS), representing a significant breakthrough in marine nitrate monitoring. The pressure-resistant miniature spectral sensor, combined with spectral deconvolution algorithms, enabled reagent-free, second-scale response and continuous in situ monitoring for the first time [17], laying the foundation for the commercialization of UV spectrophotometric nitrate sensors. Zielinski et al. designed a novel continuous observation sensor for autonomous underwater applications, such as moorings and autonomous underwater vehicles (AUVs) [18]. The sensor employed UV absorption spectroscopy combined with algorithm-based correction for seawater component interference and evaluated multiple anti-fouling strategies to mitigate biofouling effects. Field experiments conducted in the North Atlantic demonstrated its feasibility in turbid waters. Furthermore, the team developed dedicated algorithms for turbid coastal waters by using UV spectrophotometry to compensate for interference from suspended particles and dissolved organic matter. The proposed algorithm improved the robustness of nitrate concentration calculations during field measurements and demonstrated applicability in high-turbidity environments [19]. Li et al. investigated seawater nitrate determination using full-spectrum UV absorption over the 200–370 nm wavelength range [20]. This method enabled accurate, reliable, rapid, and practical nitrate measurements in seawater. Subsequently, the team developed an online seawater nitrate monitoring system based on UV spectrophotometry [21]. The system utilized UV absorption technology (190–370 nm) combined with partial least squares regression for nitrate concentration determination, eliminating the need for sample preservation and avoiding contamination issues, while offering advantages of rapid response, reliability, and high sensitivity. Nehir et al. improved the calibration and data processing procedures of the OPUS optical sensor, enhancing the resolution and accuracy of nitrate monitoring through temperature–salinity correction (TSC) algorithms and bromide interference compensation [22]. In 2022, Yu proposed a nitrate sensor design based on a deep-UV narrowband tunable light source. By employing narrowband illumination, the monochromaticity of the light source was improved, and temperature–salinity correction algorithms were developed [23]. This method achieved higher optical energy utilization efficiency than broadband light sources and reduced optical energy attenuation, although the overall system volume remained relatively large. In the same year, Dong et al. reviewed advances in in situ UV spectrophotometric detection and noted that commercial sensors (e.g., SUNA V2) still exhibited insufficient reliability and accuracy under complex environmental conditions [15]. The review highlighted challenges including light source drift and weak optical signals, and emphasized the need to improve system compactness and reduce costs in future developments. Zhang et al. developed a calibration algorithm for nitrate sensors under deep-sea pressure conditions and experimentally validated a pressure correction equation (up to a depth of 2000 m) to compensate for the pressure-induced effects on seawater UV absorption spectra [24]. This reagent-free approach is suitable for long-term in situ measurements. Arai et al. developed a temperature-compensated UV spectrophotometric method for in situ measurement of nitrate and chloride concentrations in seawater, establishing a calibration model capable of accurately estimating nitrate concentrations under varying salinity and seawater composition conditions [25]. Fernandes et al. developed a portable nitrate measurement system based on a paired UV diode–photodiode photometer, integrating a temperature sensor and quartz cuvette to achieve real-time correction of environmental interference without chemical reagents. Powered by batteries, the system enabled near-real-time field measurements [26]. In 2025, Liu et al. proposed a low-power seawater nitrate profiling method based on UV spectrophotometry [27]. By improving UV spectral measurement technology, the proposed method enabled continuous nitrate concentration profiling in seawater under low-power conditions, providing new technical support for dynamic marine nitrate monitoring.

However, for in situ monitoring applications, the aforementioned studies have not fully addressed the issues of received signal drift and attenuation caused by time-varying light source attenuation and optical energy losses within the system. To improve the measurement accuracy and detection limit of UV spectrophotometric seawater nitrate detection, while enhancing system compactness for better adaptation to in situ measurement scenarios, this paper presents a low-loss deep-sea nitrate sensing optical system based on a co-aperture coupling structure with light-source fluctuation compensation capability, incorporating a measurement path and a reference path. The principle of light source correction and the system design methodology are introduced, and the detailed design process and results are presented considering practical application requirements. Experimental validation demonstrates that the proposed system effectively compensates for measurement deviations caused by light source attenuation. Sample solution measurements and sea trial results further demonstrate that the system achieves favorable measurement accuracy and detection limit performance, satisfying the requirements for in situ deep-sea nitrate concentration monitoring.

## 2. Principle and Method

### 2.1. Principle of Light Source Fluctuation Correction

The selection of the light source is of critical importance for seawater nitrate concentration measurements using nitrate sensors. Commonly used light sources include xenon flash lamps and deuterium lamps, both of which cover the primary nitrate absorption band ranging from 200 to 400 nm. However, both types of light sources are subject to a certain degree of light intensity attenuation during operation. To mitigate the influence of such attenuation on measurement accuracy, this study employs a dual-path optical system with an all-UV configuration, in which the collimating lens and coupling lens are shared. The schematic diagram of the proposed system is shown in Figure 1.

The divergent light emitted by the light source is collimated by the collimating lens to form a parallel beam. The collimated beam is subsequently divided into two optical paths by the beam-splitting structure: one serves as the measurement path, in which the beam passes through the sample under test to generate the measurement signal; the other serves as the reference path, in which the beam bypasses the sample without interaction. The two beams are then recombined by one coupling mirror and focused onto a miniature spectrometer. A micro-shutter is integrated into the beam-splitting structure to implement a time-division multiplexing scheme, whereby the measurement path is acquired first, followed by switching to the reference path, thereby obtaining the sample measurement spectrum and reference spectrum, respectively.

After data acquisition, the spectral data obtained from both paths are processed and analyzed, and correction algorithms are applied to effectively compensate for the influence of light source attenuation on the measurement results. The overall workflow is illustrated in Figure 2.

The nitrate concentration was determined using a partial least squares regression (PLS) model [28]. The measured spectra within the wavelength range of 220–240 nm were used as the input variables. A total of 49 samples were used to establish the calibration model, while 10 independent samples were used for model validation. The optimal number of latent variables was determined based on the leave-one-out cross-validation method to minimize the prediction error and avoid overfitting. The performance of the PLS model was evaluated using the coefficient of determination (R^2^), mean absolute error (MAE), and mean absolute percentage error (MAPE).

### 2.2. System Parameter Calculation

The primary challenge in the optical design of this system is to effectively minimize optical path attenuation. The main sources of attenuation are summarized as follows: (1) The absorption spectrum of nitrate is primarily located within the ultraviolet (UV) wavelength range of 200–400 nm; however, significant optical attenuation occurs when the beam propagates through the seawater sample along the measurement path within this spectral range. (2) In the UV region, optical energy losses caused by transmission through optical components are considerably higher than those in the visible and infrared regions. (3) The optical transmission path is subject to spatial constraints, making it challenging to simultaneously optimize the collimated beam diameter and divergence angle. (4) In the dual-optical-path configuration, considerable coupling loss occurred during the injection of optical signals into the spectrometer fiber. These factors require particular consideration during the optical design process.

First, the design parameters of each component in the all-lens dual-path optical system are analyzed and calculated.

#### 2.2.1. Light Source and Collimating Lens

For the light source, the divergence angle and the source size are two critical parameters that directly influence the design of the collimating lens; therefore, they must be determined prior to the system design. Let the source divergence angle be $\theta_{1}$, the emitting source size be $d_{1}$, the collimated beam divergence angle and spot diameter be $\theta_{2}$ and $d_{2}$, respectively, and the focal length of the collimating lens be $f_{c}$. Then:

(1) $$\frac{d_{1}}{2\tan(\theta_{2}/2)}<f_{c}<\frac{d_{2}}{2\tan(\theta_{1}/2)}$$

The focal length range of the collimating lens can be calculated using the above equations. The source full divergence angle $\theta_{1}$ and the source size $d_{1}$ can be obtained from the product datasheets. The collimated beam full divergence angle $\theta_{2}$ is determined according to the system testing requirements. The collimated spot diameter $d_{2}$ should be smaller than the size of the system optical path, while the aperture of the collimating lens should be slightly larger than $d_{2}$ to avoid beam obstruction by structural components.

#### 2.2.2. Focusing Lens

The focusing lens is employed to focus the collimated beam and couple it into the spectrometer fiber. The coupling efficiency at this stage is a critical factor in the design, as it directly determines the amount of energy ultimately received by the system. The primary parameters governing the coupling efficiency of the focusing lens include its numerical aperture $NA_{r}$ and the focused spot size $d_{f}$. Let the focal length of the focusing lens be $f_{u}$ and its clear aperture be $d_{in}$, Then:

(2) $$NA_{r}=\sin(\arctan(\frac{d_{in}}{2f_{u}}))$$

Let the numerical aperture of the fiber be $NA_{f}$ and the core diameter be $d_{e}$. To achieve ideal coupling efficiency, the following conditions must be satisfied:

(3) $$NA_{r}<NA_{f}$$

(4) $$d_{f}<d_{e}$$

Satisfying Equations (3) and (4) can effectively enhance the collection efficiency of the focusing lens for the dual-path beams.

It can also be derived from Equation (3) that the focal length of the focusing lens should satisfy:

(5) $$f_{u}>\frac{d_{in}}{2\tan(\arcsin(NA_{f}))}$$

Furthermore, if the full field of view of the focusing lens is ω, The core diameter of the optical fiber at the receiving plane is $d_{r}$, then the focal length of the focusing lens should also satisfy:

(6) $$f_{u}<\frac{d_{r}}{2\tan(\omega /2))}$$

Satisfying the above equation reduces the focused spot size, thereby minimizing the coupling loss when the beam enters the optical fiber.

Furthermore, if the spot sizes of the measurement and reference beams incident on the focusing lens are $d_{in1}$ and $d_{in2}$, respectively, and the separation distance between the optical axes of the measurement and reference paths is $d_{x}$, then the following condition must be satisfied:

(7) $$d_{in}>\frac{{d_{in}}_{1}+{d_{in}}_{2}}{2}+d_{x}$$

Satisfying the above equation can avoid the coupling limitation of the focusing lens aperture on the two beams.

## 3. System Design

The design optimization of each optical subsystem was conducted based on the aforementioned principles and analytical processes. A DMT 6/10 deuterium lamp (Heraeus Noblelight, Kleinostheim, Germany) with a power of 3 W and an operational wavelength range of 200–1000 nm was selected as the light source.

To enhance light energy utilization while reinforcing compactness, the fiber-coupled output configuration was not considered for the light source. This is because optical fibers, owing to their inherent structural characteristics, are difficult to miniaturize in length. Moreover, they introduce significant transmission losses and beam obstruction. A photograph of the light source is provided in Figure 3.

Experimental measurements indicate that the light source exhibits a divergence angle of 15° with an emitting aperture of 0.3 mm after removing the fiber connector. To match the platform dimensions, optimize the sensor configuration, and ensure detection performance, the measurement path aperture was set to a minimum of 5 mm (d_2_) and a maximum of 6 mm, with a channel span of 109 mm. In general, the measurement error remains relatively small when the divergence angle of the collimated beam does not exceed 1°. Therefore, according to Equation (1), the focal length of the collimating lens is determined to be in the range of 14.3 mm to 15.8 mm. For a collimating lens employed in an illumination system, rather than an imaging system, the focal length can be reasonably controlled using a minimal number of optical elements to regulate the beam propagation trajectory. Consequently, a single-element lens was adopted as the collimator, and fused silica was selected as the substrate material to ensure a high transmittance. Furthermore, given that the source emits with a divergence angle of 15°, the numerical aperture (NA) of the collimating lens should be no less than sin(15°/2) = 0.13 to collimate all the emitted rays. Based on the aforementioned parameters, the beam does not satisfy the Lagrange invariant equality before and after collimation, and the system experiences optical energy attenuation owing to the constraints imposed by the exit aperture and collimation angle. In view of this, the collimating lens is optimized using Zemax optical design software (Zemax OpticStudio 19.4 Premium), with the optical energy utilization efficiency as the evaluation metric, to fully maximize the energy utilization efficiency of the system. The final optical layout of the collimating lens is shown in Figure 4.

The collimating lens had a focal length of 18 mm and a numerical aperture of 0.15, and the collimated output beam exhibited a divergence angle of 0.95°. The actual source parameters were configured in the non-sequential mode, and ray tracing was performed on the output beam propagating through the measurement path. The resulting detector irradiance map is shown in Figure 5.

With the incident optical power set to 1 W, the optical energy utilization efficiency of the collimating lens was calculated to be 92% by dividing the received power at the detector plane by the source power. In contrast, under identical parameters without a collimating lens, the simulated energy-utilization efficiency was only 11%. These results demonstrate that the incorporation of a collimating lens significantly enhances optical energy utilization efficiency.

When collecting the beams from the two paths, employing a separate focusing lens for each path introduces signal discrepancies owing to variations in lens characteristics while simultaneously increasing system complexity and volume. Therefore, a single focusing lens was adopted in this system to couple the beams. In this configuration, the focusing lens requires a relatively large aperture, which increases the design difficulty. To mitigate this, a beam splitter is placed after the collimating lens to divide the beam into two paths: one path (the measurement path) passes through the sample under test before reaching the coupling lens, whereas the other path (the reference path) is directed to the focusing lens via three reflective mirrors, as illustrated in Figure 6. The distance $d_{x}$ between the reference and measurement beam spots before the coupling mirror may be minimized. In the present design, $d_{x}$ was set to 0 mm.

Based on the two-beam splitting configuration, a micro-optical switch was introduced between the beam splitter and the sample under test. By controlling the on/off states of the two optical paths, dual- path signals can be acquired sequentially. Because the sensor is intended to obtain nitrate concentration profile data in the ocean, it must possess deep-sea pressure resistance. Sapphire windows (5.6 mm in thickness with a clear aperture of 12 mm) are installed on both sides of the sample cell, and in conjunction with a pressure-resistant housing, the sensor achieves a pressure rating equivalent to a water depth exceeding 4000 m. Additionally, a copper mesh is installed around the perimeter of the optical windows to prevent the adhesion of underwater contaminants, particularly biological fouling, which could otherwise degrade optical transmission.

To optimize the sensor configuration and ensure the detection performance, the maximum aperture of the reference path was set to 6 mm. Based on the beam-splitting structure shown in Figure 6, the clear aperture of the focusing lens was set to 2 × 5 = 10 mm (Clear Aperture of the First Lens: 12 mm). Furthermore, the field-of-view of the focusing lens must cover the divergence angle of the collimating lens. The spectrometer employed in this system was a ZEISS MMS UV spectrometer (Jena, Germany), which was matched with a fiber core diameter of 400 μm and a numerical aperture of 0.22 (spectral resolution of 3 nm and wavelength range of 195–400 nm). According to Equation (6), the focal length of the focusing lens was calculated to be less than 0.2/tan(0.95°/2) = 24.1 mm. Based on the aforementioned parameters, the focusing lens also fails to satisfy the Lagrange invariant, and the system experiences energy attenuation owing to the constraints of the clear aperture and fiber numerical aperture. Therefore, the focusing lens was designed and fully optimized with optical energy utilization efficiency as the evaluation metric. In the nonsequential mode, the fiber was placed at the focal position of the focusing lens, and a detector was positioned behind the fiber. The resulting optical layout of the focusing lens is shown in Figure 7.

The focusing lens had a focal length of 22.7 mm, an image-side numerical aperture of 0.22, and a total length of 52.2 mm. Along the beam propagation direction, the lens elements were composed of F_Silica, CaF_2_, and F_Silica. This material combination effectively minimizes the chromatic aberration generated by the system, which is conducive to reducing the focal-spot size. With the total incident optical power set to 1 W, ray tracing was performed, and the resulting detector irradiance map is shown in Figure 8.

As evidenced by the simulation results shown in the figure, the optical energy utilization efficiency of the single-path focusing lens reached 80%, indicating excellent focusing performance.

The aforementioned modules were assembled and integrated into the complete system, the overall appearance of which is shown in Figure 9.

The housing and the sapphire window were designed for a rated pressure resistance of 40 MPa and subjected to a pressure test at 1.25 times the rated pressure (50 MPa). The pressure was maintained for 2 h, and no leakage, structural deformation, or abnormal optical or electrical response was observed after the test. The system has a length of 519 mm and a transverse dimension of 64 mm, with a total power consumption of 6.6 W, demonstrating a compact form factor and low power consumption.

The main parameters of the system are listed in Table 1.

## 4. Experiment and Analysis

### 4.1. Verification of Light Source Correction Performance

To validate the effectiveness of the light-source correction optical path proposed in Section 2 for mitigating signal drift, verification experiments were conducted using the proposed system. First, the light source and spectrometer were activated, and pure water was introduced into the cuvette. The spectral signals of the pure water sample and reference path were then measured. The results are as follows (Figure 10):

These results serve as a reference for subsequent sample measurements. Subsequently, the spectral signals of the nitrate solution sample at a concentration of 0.2 mg/L-N and the reference path were measured, and identical measurements were repeated after a certain time interval. The obtained signals are expressed in Figure 11.

It can be observed that the signal intensities of the measurement and reference paths exhibit considerable fluctuations at 2 min and 2 h after system start-up. If these fluctuations are not corrected and the nitrate concentration is calculated directly, substantial measurement errors will be introduced. The measured signal and reference signal are differentially corrected to obtain the compensated measurement signal. The relationship satisfied by the corrected signal intensity is as follows:

(8) $$P_{C}(\lambda )=P_{M}(\lambda )-A(\lambda )\cdot P_{R}(\lambda )$$

where $P_{M}(\lambda )$ denotes the signal intensity of the measurement path at wavelength *λ*, A(λ) represents the ratio of transmittance to reflectance of the beam splitter, and $P_{R}(\lambda )$ denotes the signal intensity of the reference path at wavelength *λ*.

The signals received by the two paths were processed according to the aforementioned method, yielding both the uncorrected and corrected measurement signals, as illustrated in Figure 12.

In the figure above, the ordinate represents absorbance, denoted here as $A_{e}$, which can be expressed as:

(9) $$A_{e}=\log_{10}(\frac{I_{0}}{I})$$

where $I_{0}$ is the incident light intensity obtained from the reference path signal according to the spectral splitting ratio, and I is the intensity of the measurement path beam after passing through the sample solution. It can be observed that after correction, the absorbance curves measured by the system at different times remain stable, effectively overcoming the influence of light source fluctuations.

According to this method, additional tests were performed on nitrate solution samples with concentrations of 0.1 mg/L-N and 0.5 mg/L-N, respectively. The test results before and after the correction are as follows (Figure 13):

It can be observed that, across different concentrations, the sample absorbance curves after dual-optical-path correction all demonstrate good stability.

### 4.2. Main Performance Test

To evaluate the performance of the proposed system, key metrics including the limit of detection, precision, and accuracy were tested. The details are as follows:

#### 4.2.1. Detection Limit

The limit of detection (LOD) refers to the lowest nitrate concentration that a sensor can reliably detect. Multiple measurements were performed on the nitrate concentrations of the blank solutions, and the standard deviation σ of the measurement results was calculated using the following equation:

(10) $$SD=\sqrt{\frac{\sum_{i=1}^{n}{\left(C_{i}-M_{1}\right)}^{2}}{n-1}}$$

where $C_{i}$ denotes the *i*th measured nitrate concentration value of the blank solution, and $M_{1}$ represents the arithmetic mean of the nitrate concentration measurements of the blank solution over *n* measurements.

The limit of detection of the system can be expressed as DL=3×SD. Twenty measurements were performed on the blank solution, yielding a standard deviation of 0.0018 mg/L-N for nitrate concentration. The limit of detection was set to 0.0056 mg/L-N.

#### 4.2.2. Accuracy

Accuracy refers to the deviation between the sensor measurement results and the true values, which can reflect the precision of nitrate concentration detection. During the measurements, solutions with varying nitrate concentrations were employed as the test samples, where the nitrate concentration ranged from 0 to 5.6 mg/L-N. In this study, accuracy was evaluated using the mean absolute error (MAE), mean absolute percentage error (MAPE), and coefficient of determination (*R*^2^).

(11) $$\mathrm{MAE}=\frac{1}{n}\sum_{j=1}^{n}\left|C_{j}-S_{j}\right|$$

(12) $$\mathrm{MAPE}=\frac{1}{n}\sum_{j=1}^{n}\left|\frac{C_{j}-S_{j}}{S_{j}}\right|\times 100\%$$

(13) $$R^{2}=1-\frac{\sum_{j=1}^{n}{\left(C_{j}-S_{j}\right)}^{2}}{\sum_{j=1}^{n}{\left(S_{j}-M_{2}\right)}^{2}}$$

where $C_{j}$ denotes the measurement result at the *j*th nitrate concentration, $S_{j}$ represents the standard nitrate concentration value at the *j*th nitrate concentration, n is the total number of nitrate concentration types measured, and $M_{2}$ is the mean nitrate concentration of all standard solutions. Here, a coefficient of determination closer to 1 indicates a higher degree of agreement between the retrieved measurements and true values.

Measurements were performed on 10 nitrate solutions of different concentrations, with three replicate measurements for each concentration.

The analysis revealed that at low concentrations (0~0.64 mg/L-N), the coefficient of determination for the nitrate concentration measurements was 0.9783, with a mean absolute percentage error of 0.018 mg/L-N. At high concentrations (0.64 mg/L-N–5.6 mg/L-N), the coefficient of determination for nitrate concentration predictions was 0.9989, with a mean relative error of 3.49%.

#### 4.2.3. Precision

Precision refers to the consistency of the measurement results when a sensor measures the same sample under identical conditions. It is typically expressed as relative standard deviation (RSD).

(14) $$RSD=\frac{\sqrt{\frac{\sum_{k=1}^{n}{\left(C_{k}-C_{a2}\right)}^{2}}{n-1}}}{M_{3}}\times 100\%$$

where $C_{k}$ denotes the nitrate concentration result of the k-th measurement for the same sample, n represents the number of measurements for the same sample, and $M_{3}$ is the mean nitrate concentration obtained from the n measurements of the same sample.

Nitrate solution samples with concentrations of 0.05 mg/L-N, 0.5 mg/L-N, and 2.6 mg/L-N were prepared, respectively. Each sample was measured six times, and the predicted nitrate concentrations were retrieved for each measurement. The resulting RSD was determined to be 24.43% for the 0.05 mg/L-N nitrate solution, 4.03% for the 0.5 mg/L-N nitrate solution, and 1.01% for the 2.6 mg/L-N nitrate solution.

### 4.3. System Sea Trial

Following laboratory validation, sea trials of the proposed system were conducted. The sea trial site was located in the northern South China Sea, with a maximum deployment depth of approximately 900 m, and the system was mounted on an Argo float platform. During the experiment, the system was gradually deployed to depth, and nitrate data at different depths were collected during the ascent phase. Continuous measurements of seawater nitrate concentration profiles were performed over a one-month period, and the obtained nitrate concentration profile data at different time periods are presented in Figure 14.

It can be observed that the proposed system effectively measures nitrate concentrations at different water depths, and the measurement results are consistent with the variation trends of nitrate concentration profiles in the ocean. Meanwhile, the sea trial results further demonstrate that the system is capable of achieving long-term stable and large-depth observations of nitrate concentration profiles when deployed on deep-sea mobile platforms.

## 5. Discussion

To provide a reference for evaluating the performance of the developed nitrate sensor, its key specifications were compared with those of the commercially available Deep-SUNA nitrate sensor (Sea-Bird Scientific). The comparison results are presented in Table 2. The specifications of Deep-SUNA were obtained from the manufacturer’s publicly available datasheet.

Compared with the Deep-SUNA, a mainstream international commercial nitrate sensor, the developed system exhibits advantages in maximum pressure resistance, power consumption, and real-time compensation of light-source drift. Nevertheless, the dual-optical-path reference channel has inherent limitations. The reference optical path can effectively compensate for light-source drift, but it cannot compensate for changes in optical-window transmittance caused by biofouling or pressure, or for contamination of the sample cell. These factors may introduce additional measurement uncertainty during long-term in-situ deployment in complex marine environments.

It should also be noted that the detection limit obtained using prepared standard solutions may not be directly equivalent to the detection performance in natural seawater. The presence of bromide in seawater, which has absorption in the deep-UV region, may introduce additional interference in nitrate determination. Therefore, the detection limit obtained under standard-solution conditions may be better than that under actual seawater conditions.

Therefore, extended long-duration sea trials will be conducted in future studies to further evaluate the performance of the developed system under real in-situ marine conditions, including its detection limit, measurement accuracy, and long-term drift. Comparative measurements with mainstream commercial sensors will also be performed under the same marine conditions to further assess the reliability and applicability of the developed system for long-term ocean observations.

## 6. Conclusions

In order to minimize the attenuation influence of the light source in the nitrate sensor, improve measurement accuracy, and enhance the optical energy transmission efficiency and compactness of the system, this paper, starting from the design principles and methodologies, first elaborates on the characteristics of the light source correction optical path of the low-loss nitrate sensor and the calculation methods for system parameters, and then describes the design results of the major system modules, including the collimating lens, beam-splitting system, and focusing lens. Through optimized design, the energy attenuation of the output light from the collimating lens was 81 percentage points lower than that before collimation, and the coupling efficiency of the single-path coupling lens reached 80%. On this basis, laboratory tests were conducted to evaluate the light source correction performance of the system. Compared with the uncorrected condition, the signal drift was reduced and the measurement stability was markedly enhanced. Performance evaluation results indicate that the detection limit of the system reaches 0.0056 mg/L-N, with satisfactory accuracy and precision. Furthermore, sea trials were carried out, and the results demonstrate that the system is capable of achieving long-term, effective, and continuous observation of deep-sea nitrate concentration profile data. This study is expected to make a significant contribution to the advancement of marine sensing technology as well as the research and remediation of ecological issues caused by nitrate in seawater.

## Figures and Tables

**Figure 1:**
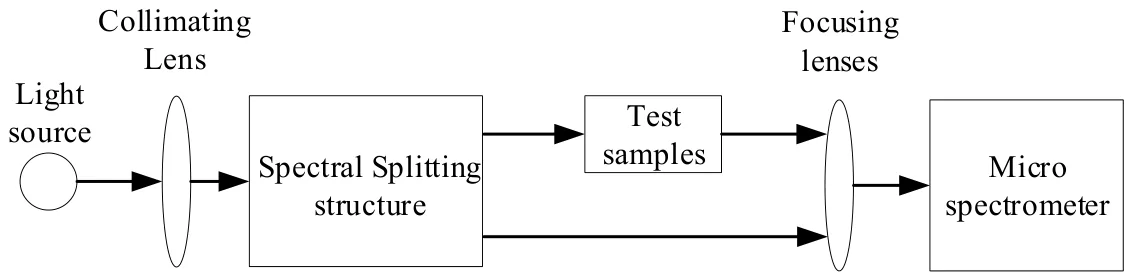
Schematic diagram of double optical path nitrate measurement system.

**Figure 2:**
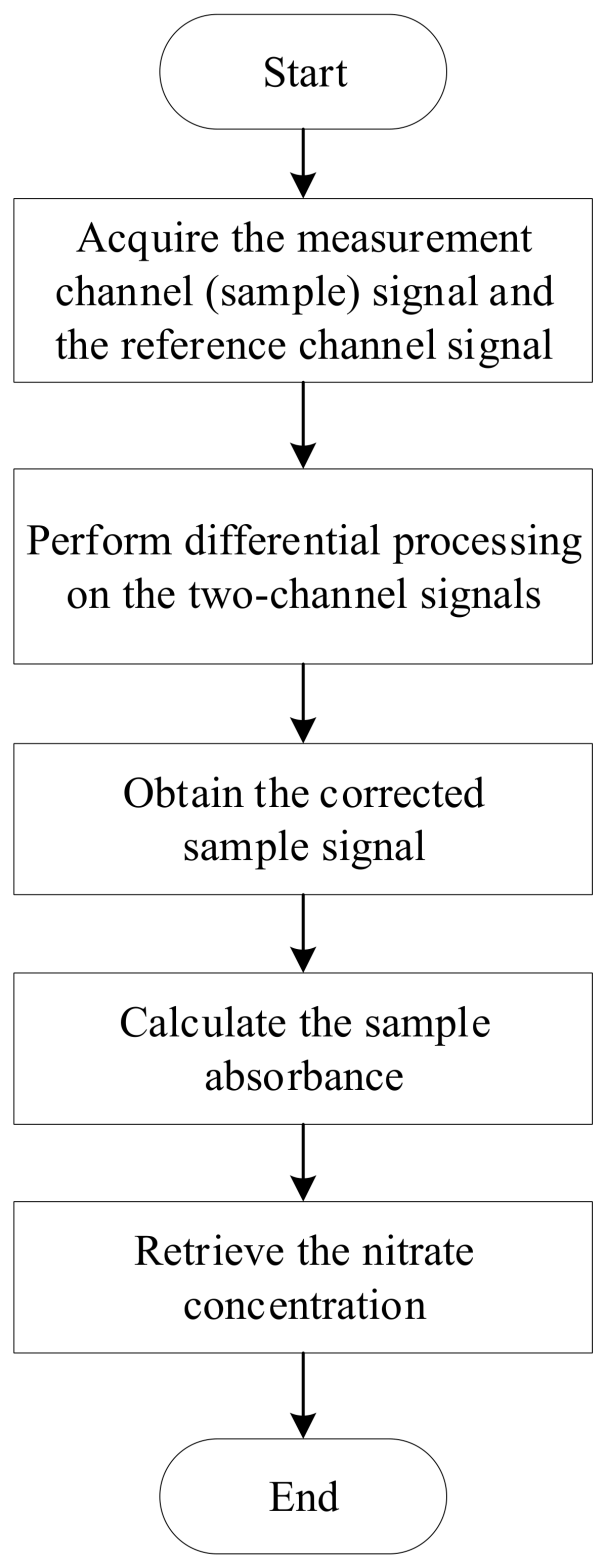
System workflow chart.

**Figure 3:**
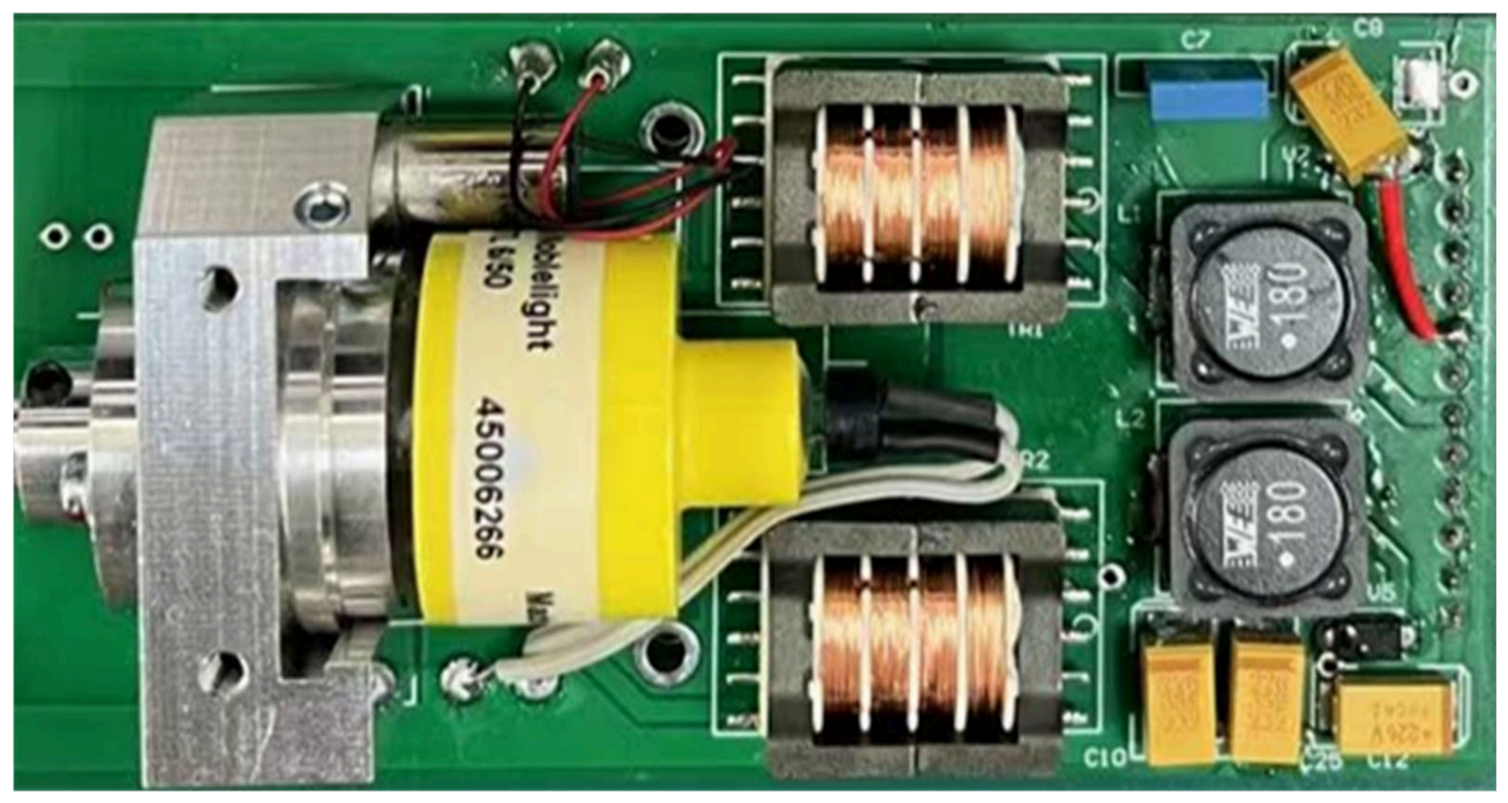
Physical drawing of the light source.

**Figure 4:**
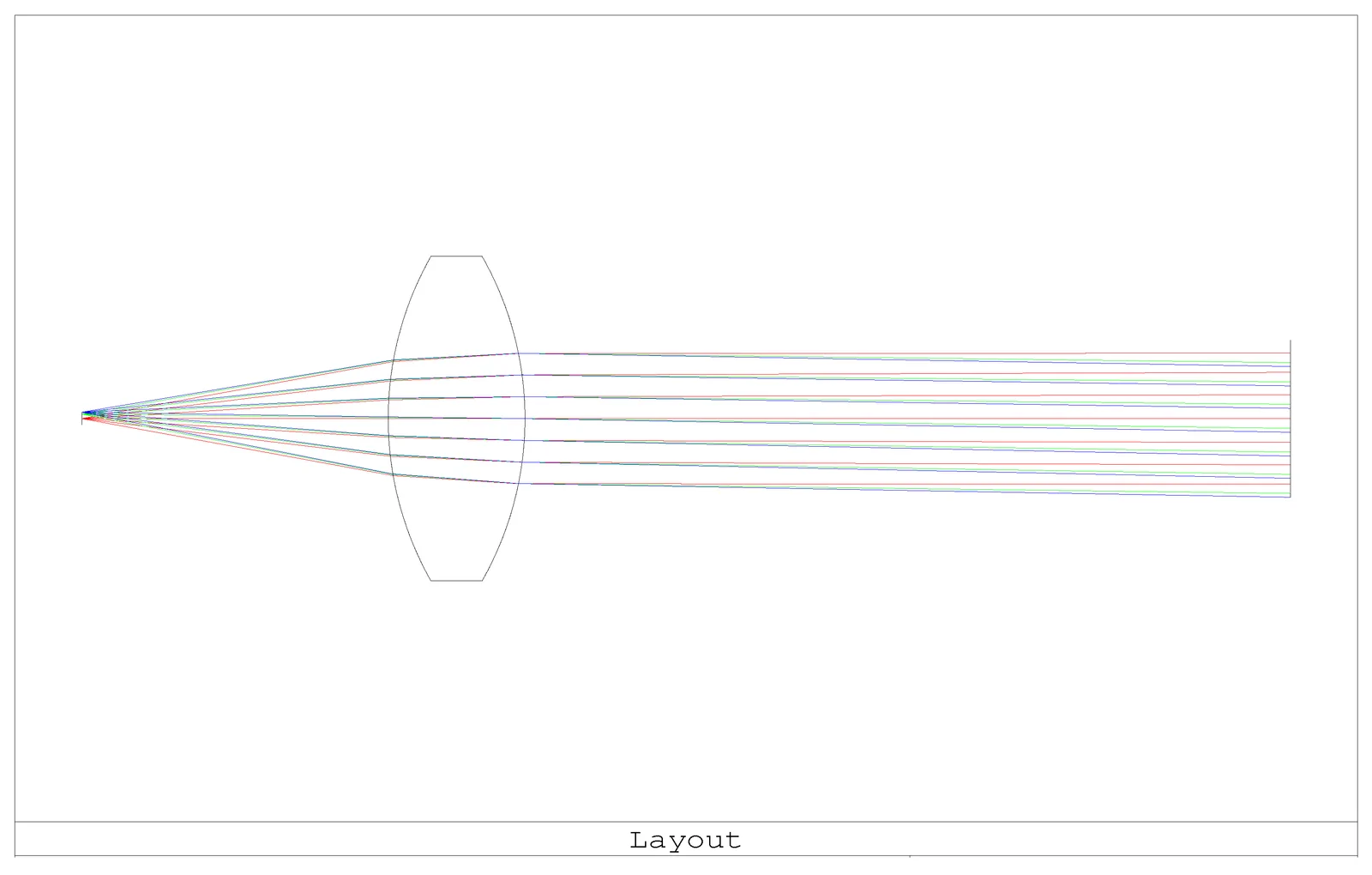
Optical path of the collimating lens.

**Figure 5:**
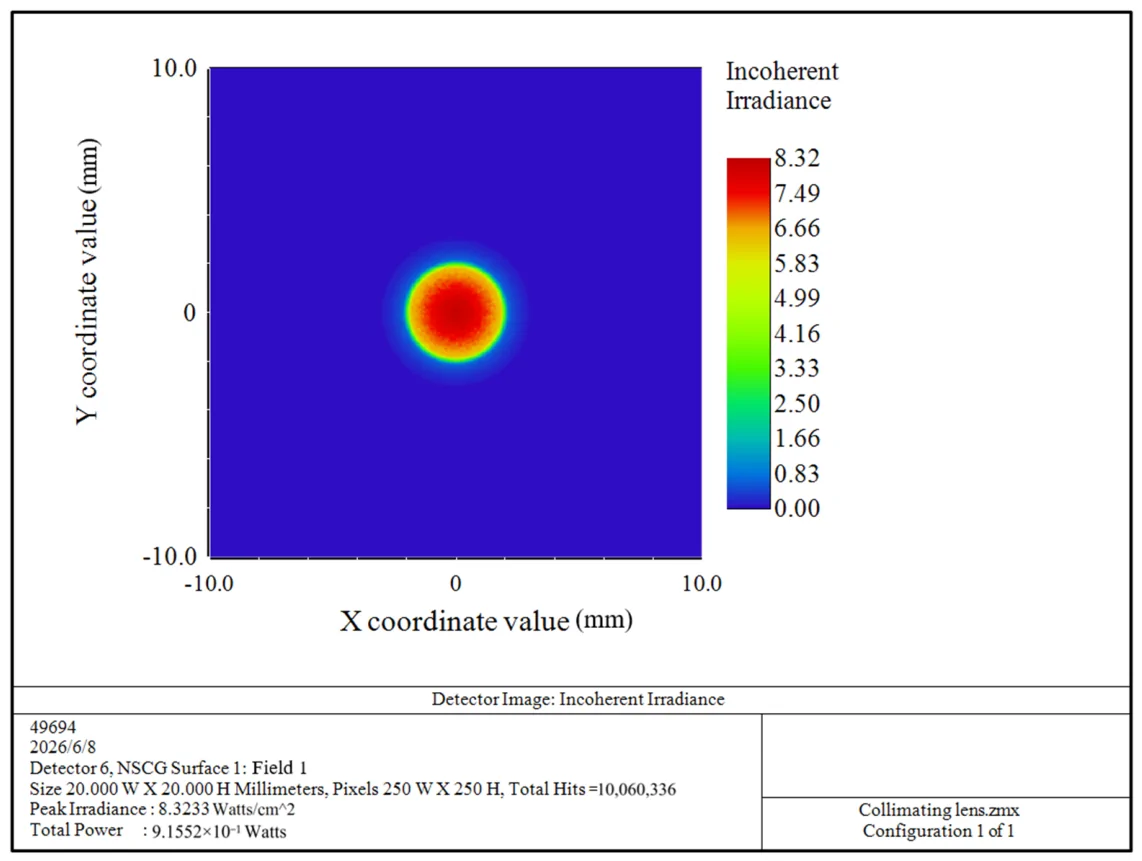
Simulation diagram of collimated light irradiation effect.

**Figure 6:**
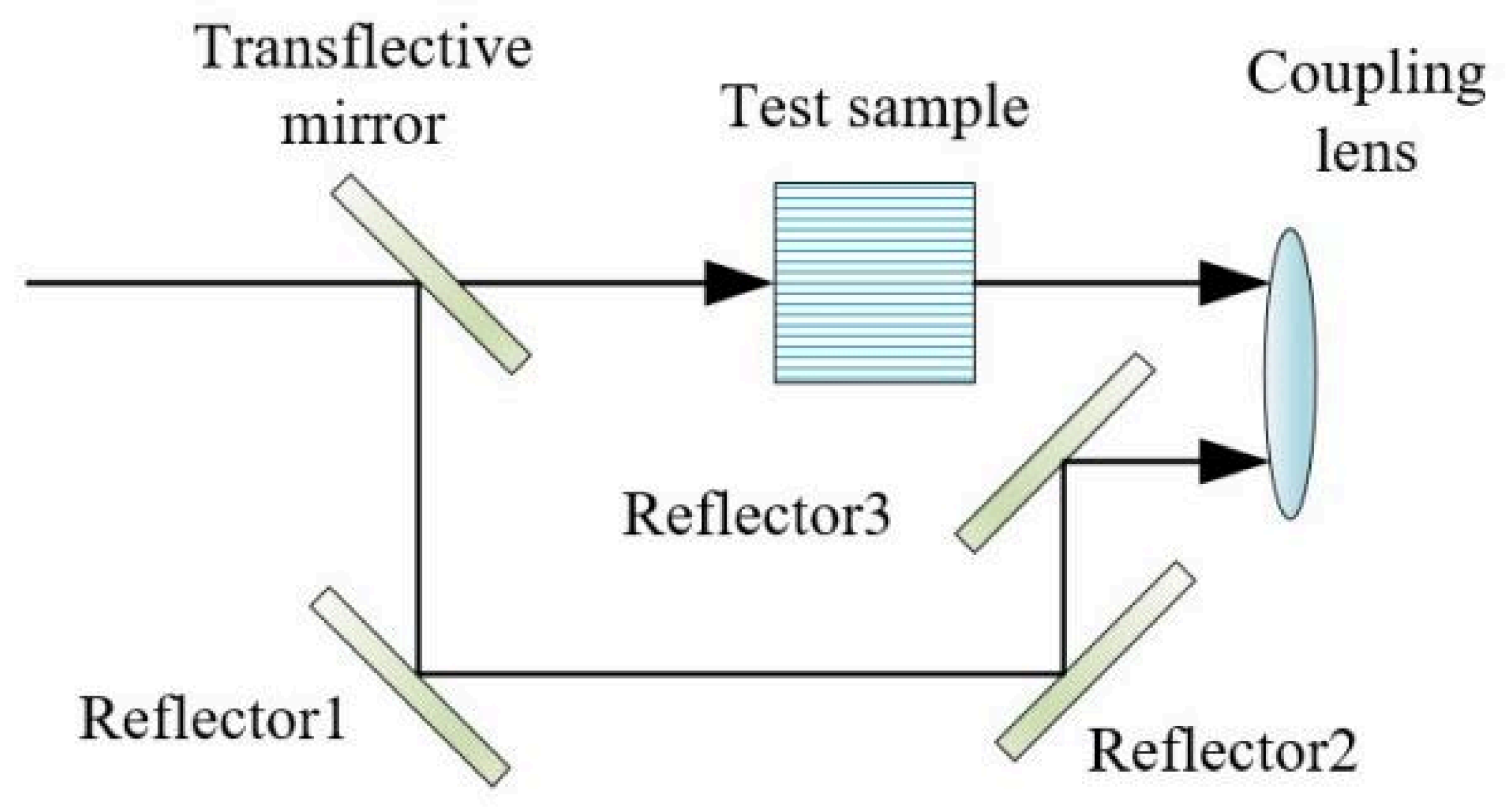
Schematic diagram of the beam splitter.

**Figure 7:**
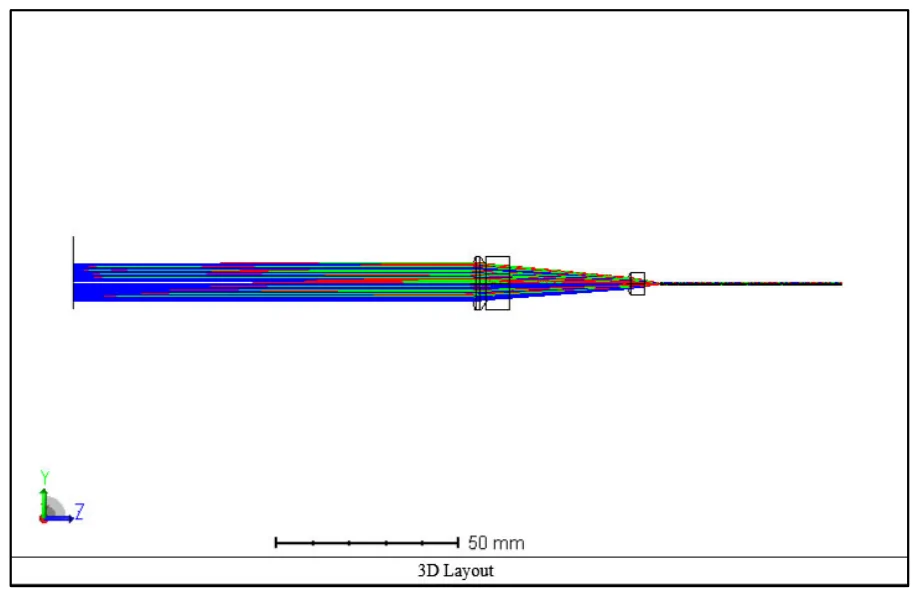
Optical path of the focusing lens.

**Figure 8:**
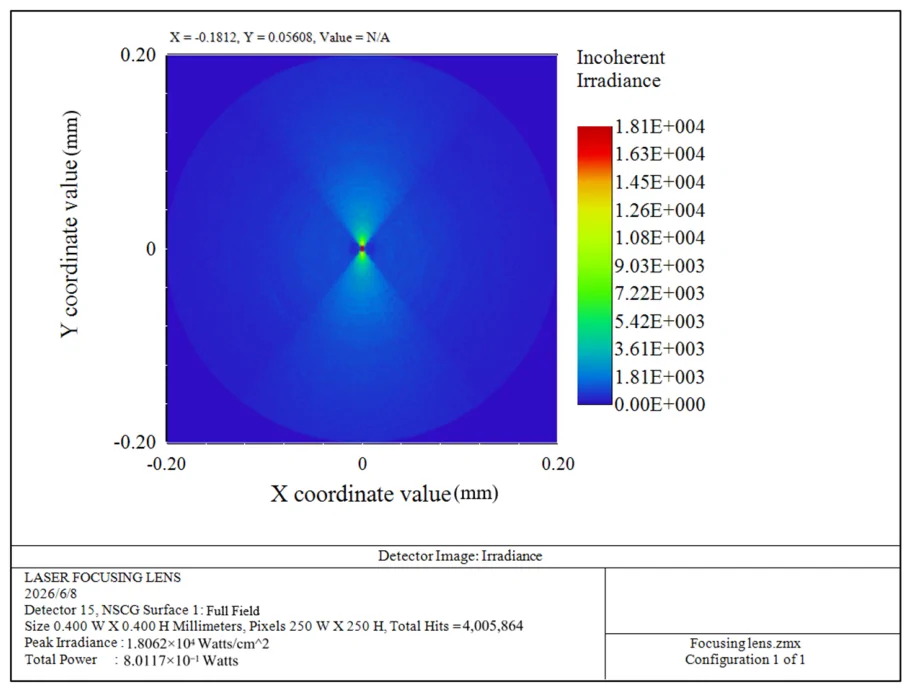
Simulation diagram of focusing lens coupling light energy.

**Figure 9:**
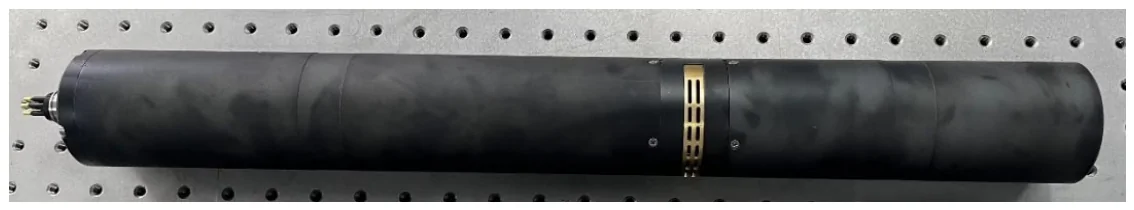
Appearance of nitrate sensor.

**Figure 10:**
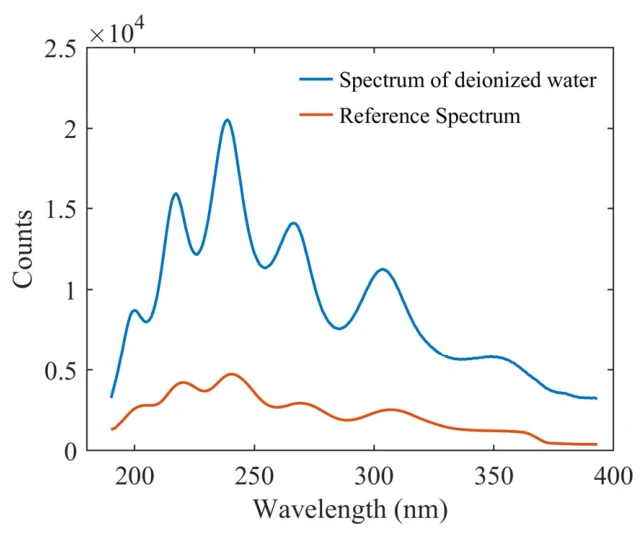
Received signals of pure water and reference path.

**Figure 11:**
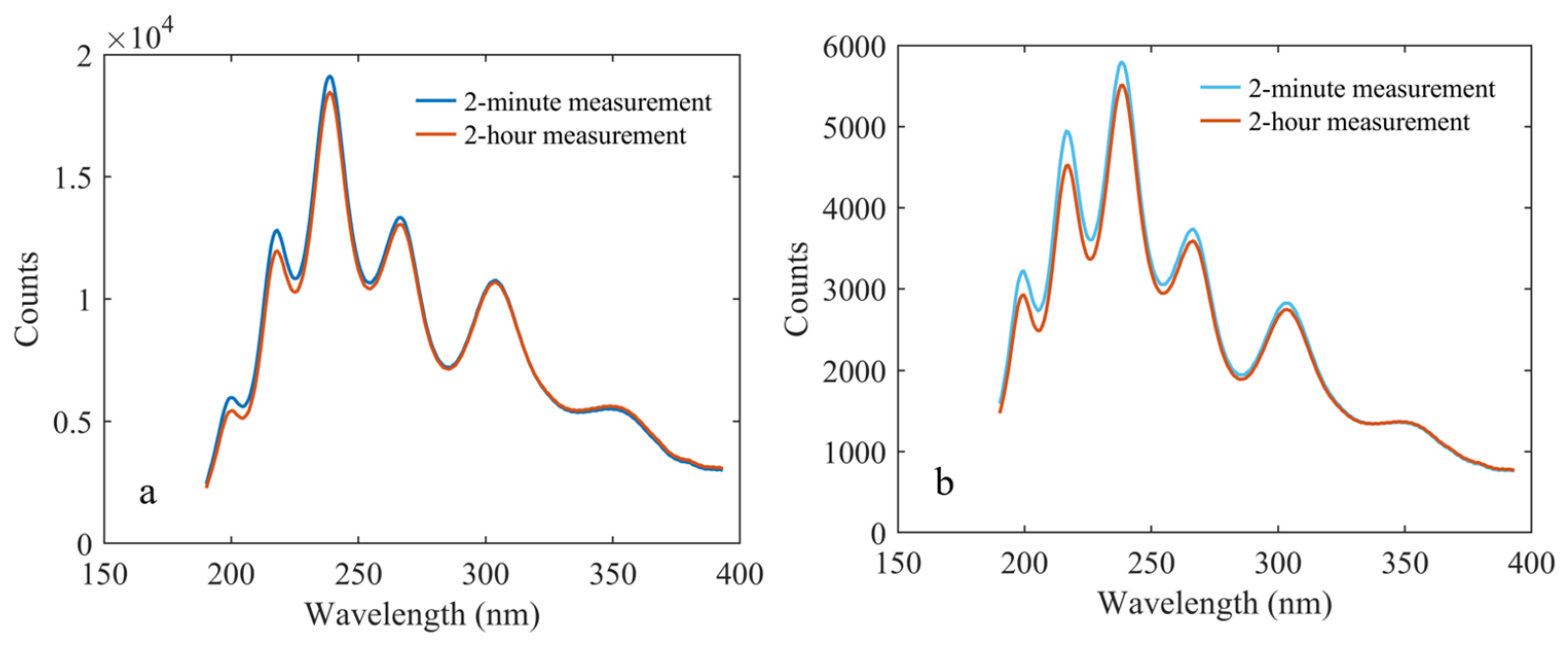
Signals received by the measurement and reference optical paths at 2 min and 2 h after instrument startup ((**a**): measurement path, (**b**): reference path).

**Figure 12:**
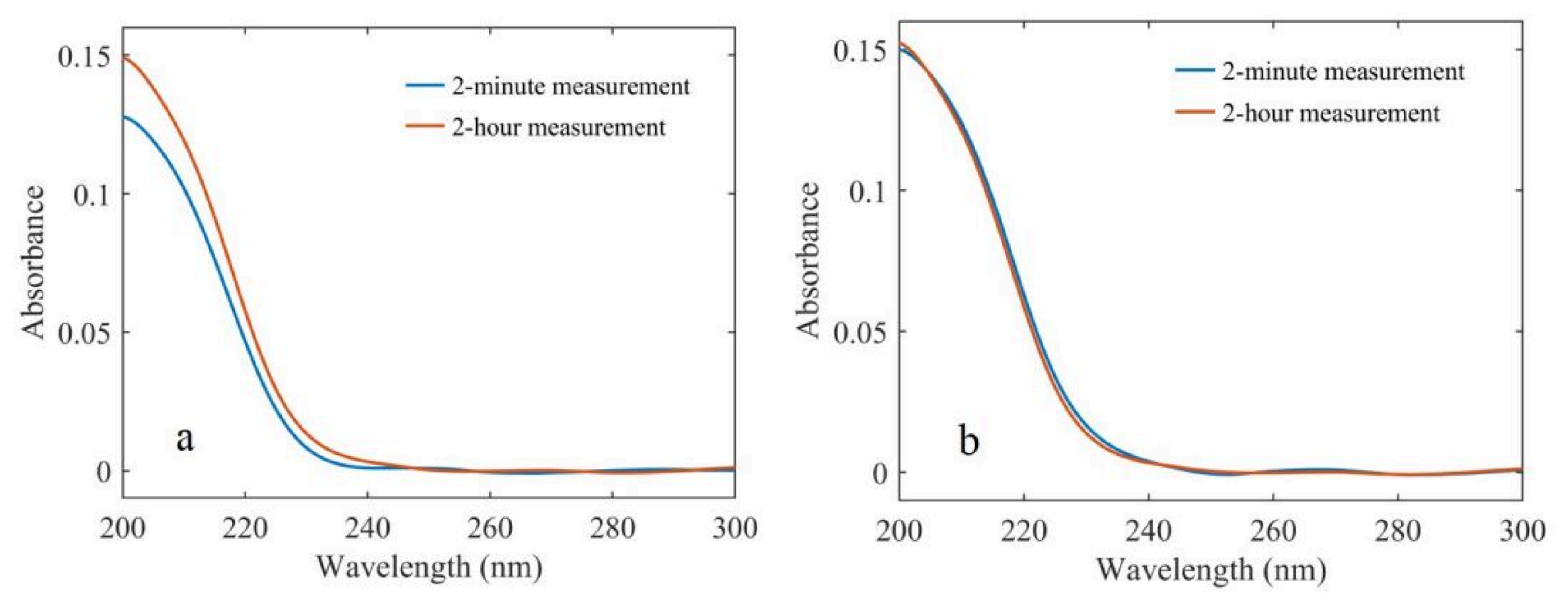
Absorbance curves of the sample at 2 min and 2 h after instrument startup ((**a**): Uncorrected, (**b**): Corrected).

**Figure 13:**
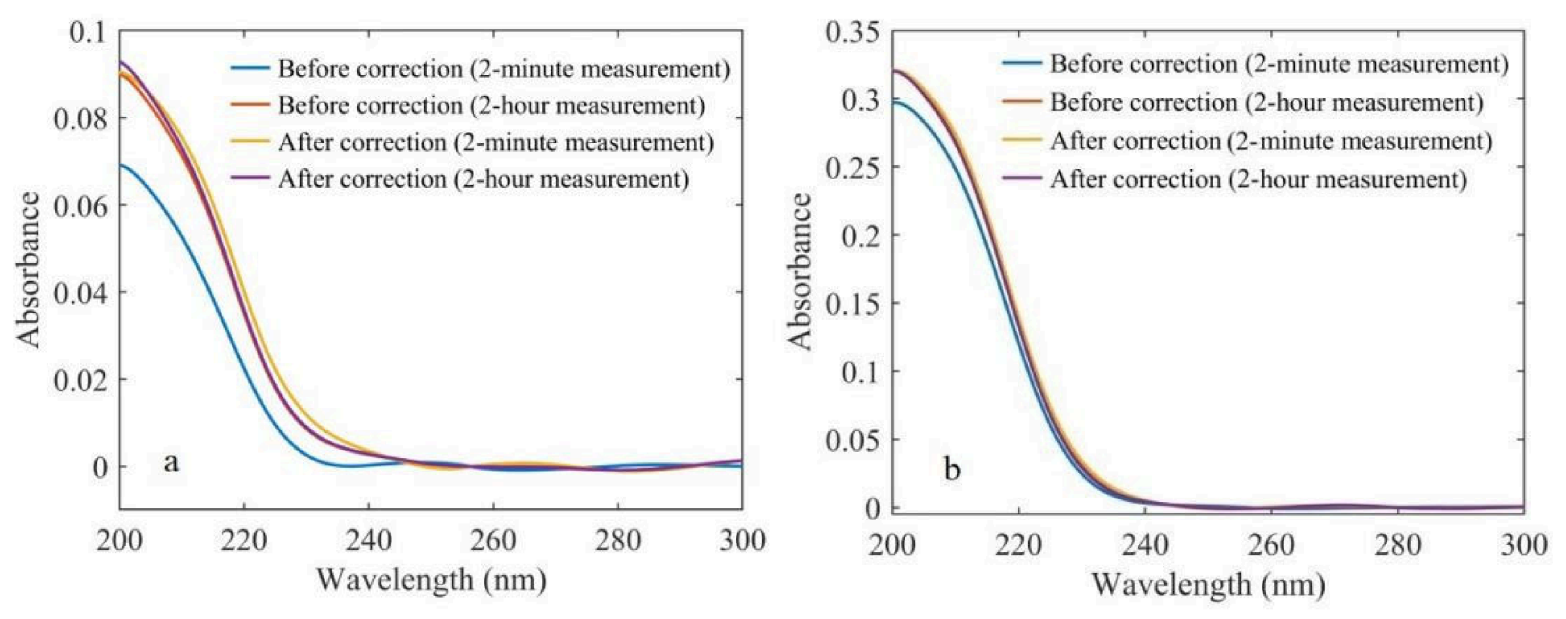
Absorbance of samples with other concentrations and the reference path before and after calibration at 2 min and 2 h after instrument startup ((**a**): Concentration: 0.1 mg/L-N, (**b**): Concentration: 0.5 mg/L-N).

**Figure 14:**
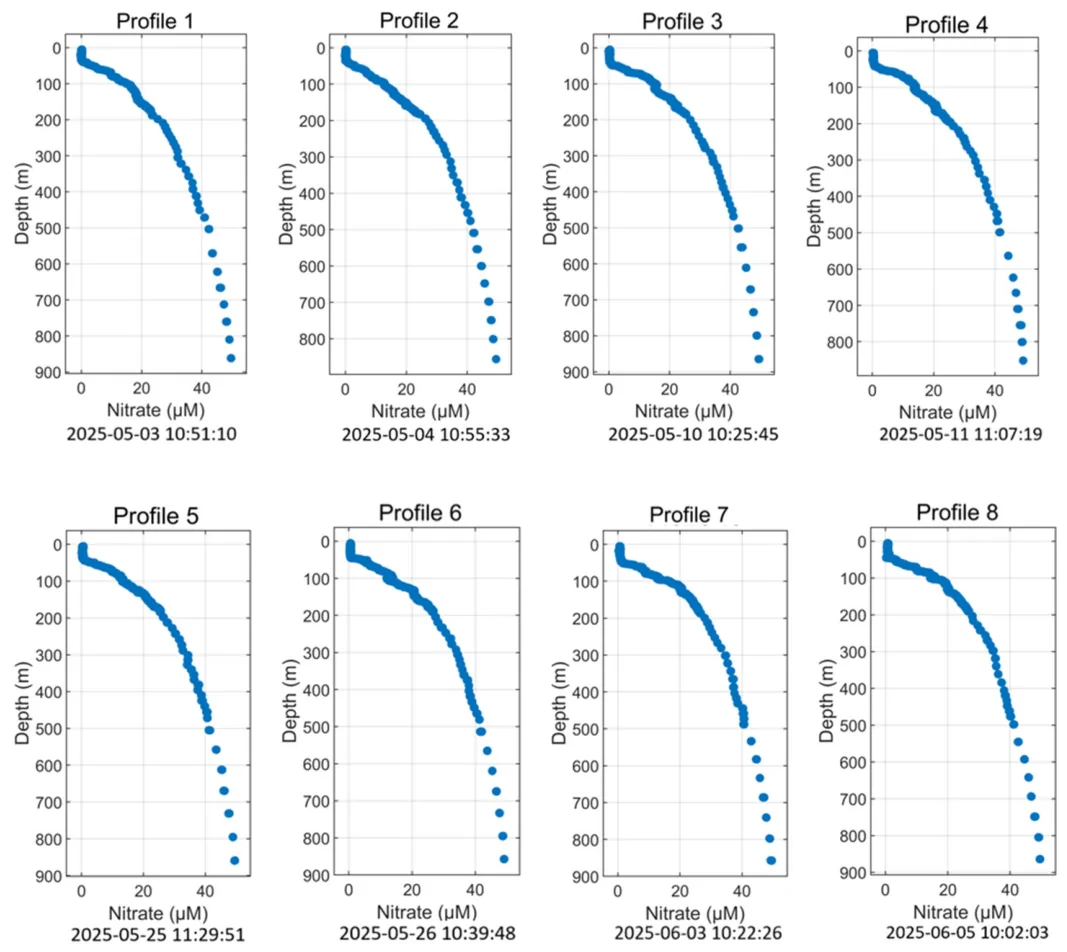
Test results of deep-sea nitrate concentration profiles of this system at different time periods. Nitrate concentration is expressed in µM, with 1 µmol/L ≈ 0.014 mg/L-N.

**Table 1:** List of Main System Parameters.

Parameter	Value	Unit
Light Source	DMT 6/10 deuterium lamp	—
Light Source Power	5	W
Operating Wavelength	200–1000	nm
Spectrometer	ZEISS MMS UV	—
Spectral Resolution	3	nm
Collimating Lens Focal Length	15	mm
Collimating Lens NA	0.18	—
Focusing Lens Focal Length	22.7	mm
Focusing Lens NA	0.22	—
Optical Fiber Core Diameter	400	μm
Sample Cell Optical Path	10	mm
Effective Clear Aperture	10	mm
System Length	519	mm
System Width	64	mm
Total Power Consumption	<6	W
Pressure Resistant Depth	>4000	m
Collimation Efficiency	92 (92, measured value)	%
Coupling Efficiency	80 (79, measured value)	%

**Table 2:** Comparison of the key performance specifications of the developed nitrate sensor and Deep-SUNA.

Indicator Name	The Sensor in this Paper (Prepared Solution)	Deep SUNA (Seawater)
Detection Limit	0.0056 mg/L-N	0.007 mg/L-N
Accuracy	0.028 mg/L-N	0.028 mg/L-N
Precision	0.013 mg/L-N	0.0042 mg/L-N
Pressure Resistance Depth	4000 m	2000 m
Power Consumption	6.6 W	7.5 W
Correction Strategy	Dual Optical Path Differential	Regular Calibration Data Correction

## Data Availability

The data used to support the findings of this study are available from the corresponding author upon request.
